# Supplementary material for: Inhibition of cell proliferation does not slow down echinoderm neural regeneration
Source: Front Zool. 2017 Feb 23;14:12. doi: 10.1186/s12983-017-0196-y (PMC5324207; doi:10.1186/s12983-017-0196-y)
Supplement: Additional file 1 — Data from two pilot experiments that were carried out to determine the dosage of aphidicolin and injection schedule. (PDF 447 kb) [file 12983_2017_196_MOESM1_ESM.pdf]

Determination of optimal aphidicolin injection parameters

Experiment I: 2 injections, 2.5 μg/g

There were two groups of animals (3 animal per group) treated with:

- aphidicolin in DMSO 2.5 μg/(g body weight) ($\sim 7.4\mu$M)
- DMSO alone: 0.25 μl/(g body weight) (0.025%)

The concentrations above are final concentrations of the drug in the animal.

The effect of aphidicolin was tested during the growth phase of the regenerating radial nerve cord (RNC). The first injection was done on day 11 post-injury followed by the second injection on the next day (day 12 post-injury). Four hours after the second injection, the animals were be fixed for immunohistochemistry. BrdU (50 mg/kg) was co-injected with aphidicolin or DMSO.

OUTCOME: The abundance of BrdU-incorporated cells decreased by $\sim$53% in aphidicolin-treated animals

Table 1: Ratio of $BrdU^+$ cells to the total cell number

	**Control, %**	**Aphidicolin 2.5μg/g**, %
Animal#1	10.76	5.88
Animal#2	27.77	4.67
Animal#3	21.61	18.10
**MEAN:**	20.05	9.55
STDEV:	8.61	7.43
SE:	4.97	4.29

t-test p-value = 0.1866

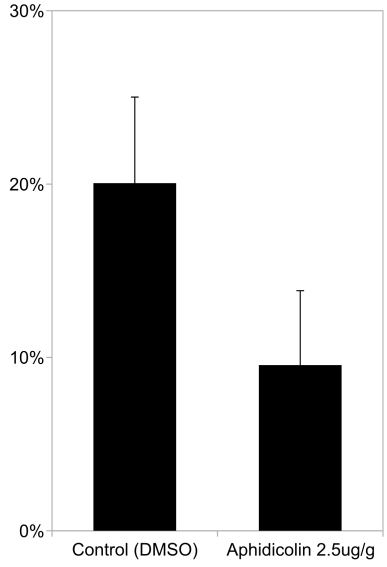

Experiment II: 7 injections, 8.3 μg/g

The following changes were made in the protocol:

- The dosage of aphidicolin was increased to 8.3 μg/g (or 25 μM).
- The number of injections was increased to 7 (one injection per day). The first injection was performed on day 6 post-injury, and the last injection on day 12 post-injury. The very first injection (on day 6), delivered the inhibitor alone, all subsequent injections delivered both the inhibitor and BrdU 50 mg/kg. The animals will be sacrificed 24 hours after the last injection.

OUTCOME: The abundance of BrdU-incorporated cells decreased by $\sim$59% in aphidicolin-treated animals

Table 2: Ratio of $BrdU^+$ cells to the total number of cells

	**Control, %**	**Aphidicolin 8.3μg/g, %**
Animal#1	18.66	4.63
Animal#2	27.78	13.63
Animal#3	22.96	9.97
**MEAN:**	23.13	9.41
STDEV:	4.56	4.53
SE:	2.63	2.62
t-test:	p-value = 0.021*	

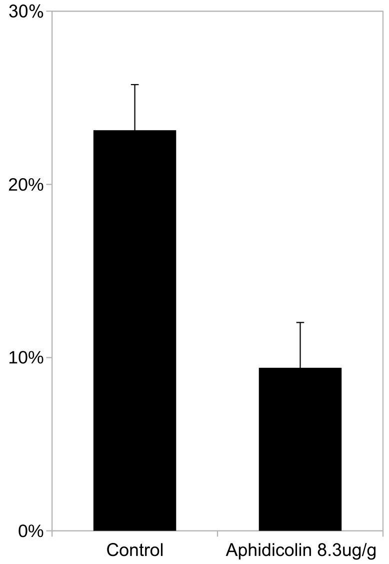

Surprisingly, the increase in the concentration of the inhibitor and in the number of injections did NOT result in a more extensive suppression of the number of $BrdU^+$ cells, only the individual variation in response to the treatment became much smaller, so that the difference is now statistically significant.

For the final experiment, which is reported in the main text of the paper, we decided to stay with the same dosage (8.3 μg/g), but increase the number of injections to 15, to cover the entire period of regeneration between days 1 and 15 post-injury, i.e. from the early post-injury phase thru the growth phase, when the animals were sacrificed for analysis.
