# Supplementary figures and images for: Inhibition of cell proliferation does not slow down echinoderm neural regeneration
Source: Front Zool. 2017 Feb 23;14:12. doi: 10.1186/s12983-017-0196-y (PMC5324207; doi:10.1186/s12983-017-0196-y)

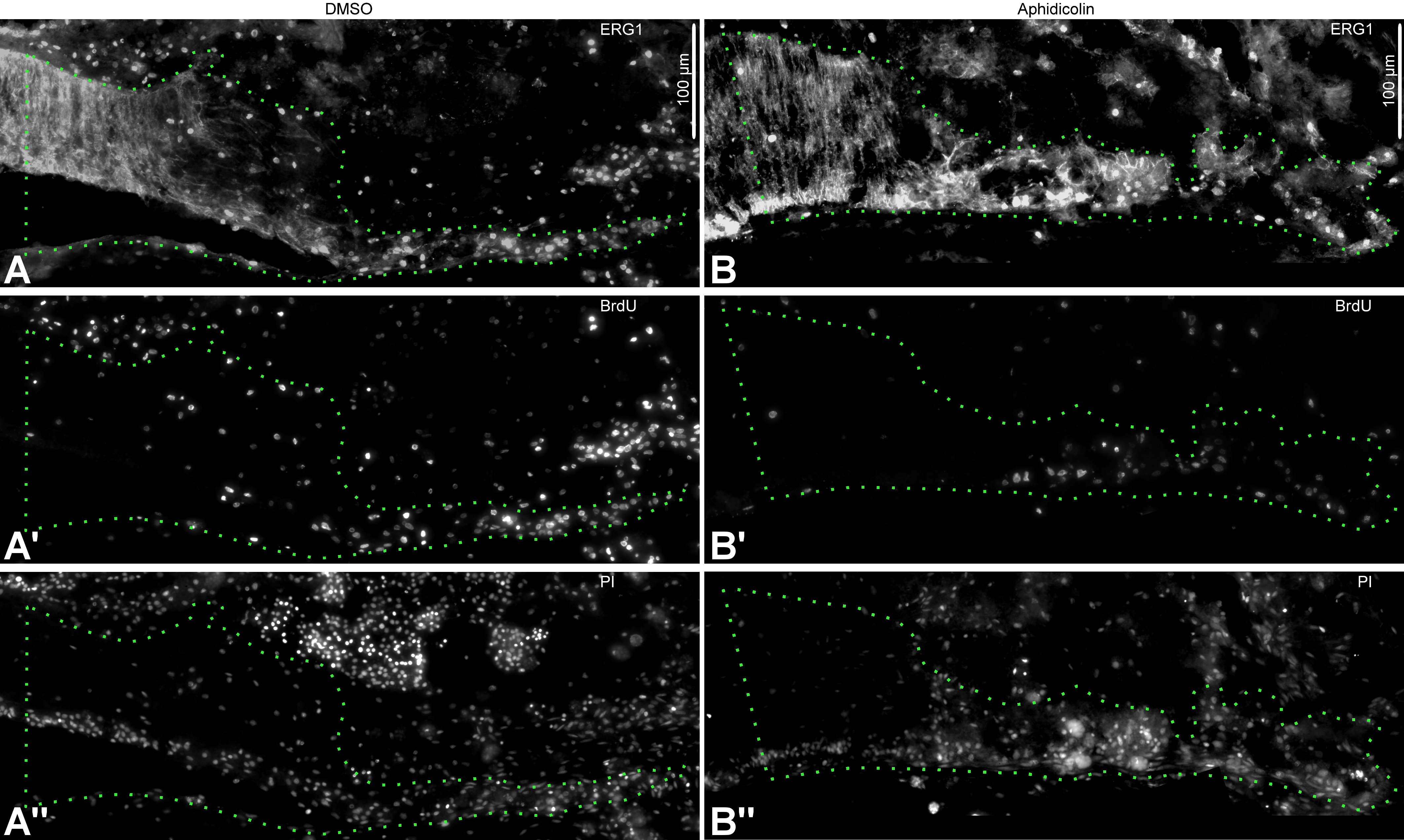

Supplement: Additional file 4 — The same micrographs as in Fig. 1E, F shown in three separate fluorescent channels (A) – (A”) Regenerating RNC in a control animal (corresponds to Fig. 1E). (B) – (B”) Regenerating RNC in an aphidicolin-treated animal (corresponds to Fig. 1F). The top row shows ERG1 antibody staining; the second row shows BrdU immunocytochemistry; and the bottom row shows nuclear staining with propidium iodide (PI). The dotted lines mark the ares of the RNC where the cells were counted (regenerate + 100 μm of the proximal stump tissue). (JPG 2846.72 kb) [file 12983_2017_196_MOESM4_ESM.jpg]
